# The effects of romosozumab combined with active vitamin D_3_ on fracture healing in ovariectomized rats

**DOI:** 10.1186/s13018-022-03276-1

**Published:** 2022-08-12

**Authors:** Ryota Takase, Yuta Tsubouchi, Takefumi Otsu, Takashi Kataoka, Tatsuya Iwasaki, Masashi Kataoka, Hiroshi Tsumura

**Affiliations:** 1 Oita University Hospital Rehabilitation Center, Oita University, 1-1 Idaigaoka, Hasama-machi, Yufu-city, Oita, 879-5593 Japan; 2 School of Physical Therapy, Faculty of Rehabilitation, Reiwa Health Sciences University, 2-1-12 Wajirogaoka, Higashi-ku, Fukuoka, 811-0213 Japan; 3 Division of Mechatronics, Department of Innovative Engineering, Faculty of Science and Technology, Oita University, 700 Dannoharu, Oita, 870-1192 Japan; 4 Department of Rehabilitation Medicine, Faculty of Medicine, Oita University, 1-1 Idaigaoka, Hasama-machi, Yufu-city, Oita, 879-5593 Japan; 5 Physical Therapy Course of Study, Faculty of Welfare and Health Sciences, Oita University, 700 Dannoharu, Oita-city, Oita, 870-1192 Japan; 6 Department of Orthopaedic Surgery, Faculty of Medicine, Oita University, 1-1 Idaigaoka, Hasama-machi, Yufu-city, Oita, 879-5593 Japan

**Keywords:** Romosozumab, Fracture healing, Ovariectomy, Vitamin D

## Abstract

**Background:**

In this study, we investigated the potential acceleration of fracture healing and bone mineral density-increasing effects of romosozumab and active vitamin D_3_ combination therapy for fractures in ovariectomized rats.

**Methods:**

Ovariectomy was performed on 40 24-week-old female Sprague–Dawley rats. After 8 weeks, the rats were subjected to periosteum removal and osteotomy of the femoral shaft followed by osteosynthesis with intramedullary nailing to create fracture models. The rats were then divided into four groups: C group (control), R group (receiving romosozumab at 25 mg/kg once a month via subcutaneous injection), VD group (receiving active vitamin D_3_ at 0.2 µg/kg twice a week via subcutaneous injection), and R + VD group. Further, 10 rats were included in a sham group. At 10 weeks after the intervention, both femurs were removed and blood samples were collected from all rats. Soft X-ray imaging was used to evaluate bone union, and microcomputed tomography (micro-CT) was used for bone morphometric evaluation. Toluidine blue staining was used for the histopathological evaluation of the undecalcified specimens, and bone turnover marker levels were measured using enzyme-linked immunosorbent assay.

**Results:**

Bone morphometry analysis via micro-CT revealed increased mineral density of the trabecular bone in the R + VD group femurs, demonstrating the effectiveness of romosozumab plus active vitamin D_3_ combination therapy. However, there were no differences in bone union evaluated using soft X-ray imaging, indicating no acceleration of fracture healing.

**Conclusions:**

Although romosozumab and active vitamin D_3_ combination therapy increased trabecular bone volume, there was no evidence on its ability to accelerate fracture healing.

**Graphical abstract:**

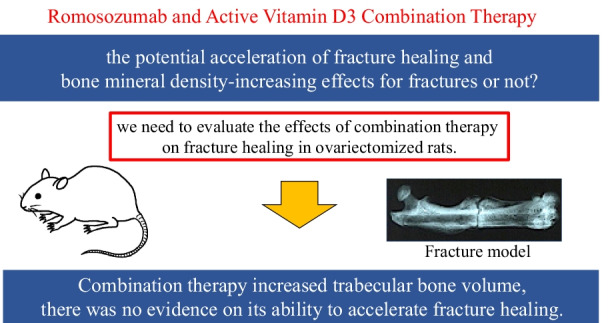

## Background

The incidence of delayed union and nonunion in all fractures is approximately 5% [1, 2]. When these complications occur after osteosynthesis, they can occasionally result in considerable functional and socioeconomic problems and can severely affect activities of daily living in patients; therefore, achieving early bone union and ensuring the return patients to society are important issues [3, 4].

Romosozumab is an antibody that targets sclerostin and is useful for treating osteoporosis, especially in elderly women with severe osteoporosis.

Sclerostin is secreted by osteocytes in the bone microenvironment and can inhibit the canonical Wnt (cWnt)/β-catenin pathway [5–7]. The cWnt/β-catenin pathway plays an important role in bone remodeling and is activated when the Wnt protein binds to the receptor complex comprising the frizzled receptor and low-density lipoprotein receptor-related protein (LRP) 5 or LRP 6, which are co-receptors of the LRP family [8]. Osteocytes are crucial for the regulation of the cWnt/β-catenin pathway [9]. One of the regulation mechanisms involves the secretion of sclerostin that inhibits Wnt signaling. Sclerostin binds to the Wnt co-receptor LRP5/LRP6 and inhibits co-localization with the frizzled receptors [5–7]. When the stoichiometry levels of sclerostin exceed that of the Wnt ligand, the signals will not be activated, thus leading to β-catenin degradation, lower bone formation, osteoblast genesis, and higher bone resorption for increasing the levels of receptor activator of NF-κB ligand [10].

Several reports have described the effect of romosozumab on promoting bone healing in animal models [11–13]. In contrast, romosozumab was found to increase the callus volume but not bone healing [14, 15].

We previously reported that a combination of low-dose teriparatide and zoledronic acid promotes callus formation, increases callus volume, and enhances bone union in a refractory fracture rat model [16]. Teriparatide is an anabolic agent that stimulates bone formation. Romosozumab also has an anabolic effect on bone but involves a different mechanism of action. Teriparatide increases bone formation and resorption via PTH–PTH receptor signaling. In contrast, romosozumab promotes bone formation through cWnt/β-catenin signaling [17] while simultaneously decreasing bone resorption. This difference indicates that teriparatide-mediated promotion of bone formation is based on remodeling, whereas romosozumab-mediated promotion of bone formation is based on modeling [18, 19]. At present, we need to evaluate the effects of romosozumab on fracture healing. Co-administration of active vitamin D_3_ is recommended with romosozumab. Active vitamin D_3_ administration is essential because reducing osteoclast activity can lead to hypocalcemia.

To date, no study has reported the acceleration of fracture healing after romosozumab and active vitamin D_3_ combination therapy in ovariectomized (OVX) rat fracture models. Therefore, we hypothesized that an excessive decrease in osteoclast activity after romosozumab administration should inhibit endochondral ossification bone union in a fracture model, with the hypocalcemia induced by romosozumab administration producing an additional negative effect on bone fusion. To test this hypothesis, we investigated the promotion of fracture healing effect of romosozumab and active vitamin D_3_ combination therapy in an OVX rat fracture model.

## Methods

### Surgical techniques used for ovariectomy

Ethical approval was obtained from Oita University’s Animal Research Committee prior to animal experimentation (Oita University Institutional Animal Ethics Committee, no. 182402). A total of 40 female Sprague–Dawley rats (24 weeks old; CLEA Japan, Inc., Tokyo, Japan) were anesthetized via intraperitoneal injection with 0.3–0.4 ml of 0.15 mg/kg medetomidine + 2 mg/kg midazolam + 2.5 mg/kg butorphanol. All operations were conducted in a standard sterile environment. The rats were placed in the supine position, the abdomen was disinfected, and an approximately 2-cm skin incision was made on the median abdomen using a scalpel. The peritoneum was incised and spread using forceps and tweezers to identify the subperitoneal right ovary. Fat was grasped using hookless tweezers and pulled out through the incision, and the ovary, oviduct, and part of the uterus were elevated. The oviduct and blood vessels under the ovary were ligated using a silk thread, and the ovary was removed. After ovariectomy, the proximal side of the transfused oviduct was returned to the peritoneum. The left ovary was removed in a similar manner. Finally, the peritoneum and epidermis were ligated and sutured using a 3-0 nylon thread. In the sham group, the ovaries were pulled out in the same manner. The organs were then replaced in the peritoneum without performing ovariectomy. The peritoneum and epidermis were ligated and sutured using a 3-0 nylon thread. OVX and sham group rats were raised for 8 weeks, and their weight was measured to confirm weight gain.

### Surgical techniques used to construct the femoral fracture model

After 8 weeks, all rats underwent right hind limb femoral osteotomy. The rats were in the lateral position, and, using the posterolateral approach, their right femur was located. The periosteum of the femur was circumferentially incised, elevated, and stripped. Subsequently, the femur at the osteotomy site was exposed and transverse osteotomy was performed using a sagittal saw (Stryker, Kalamazoo, MI, USA) without cooling at the midshaft of the femoral bone. Fracture fragments were contacted and stabilized, and the fracture was fixed using a stainless-steel wire (diameter, 1.4 mm). The wire was cut on the surface of the intercondylar groove to avoid restriction of knee joint motion. Fascial and skin incisions were closed using a 3-0 nylon suture. During surgery, a mixture of medetomidine + midazolam + butorphanol was administered as anesthesia; medetomidine and butorphanol have strong analgesic effects. The rats were housed in separate cages, given food and water ad libitum, and their conditions were monitored daily.

### Study groups

**Fig. 1:**
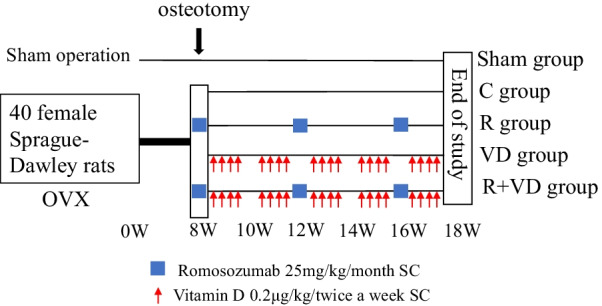
Study schema. Control group (C: administered saline), romosozumab group (R: subcutaneous romosozumab injection 25 mg/kg/once a month), vitamin D_3_ group (VD: subcutaneous vitamin D_3_ injection 0.2 µg/kg/twice a week), R + VD group. Following ovariectomy, 40 female Sprague–Dawley rats (aged 24 weeks) were divided into four groups (*n* = 10 in each group). After 8 weeks, femoral osteotomy was performed. At 18 weeks, rats were killed, and femoral bone and blood samples were taken

All 40 operated rats were divided into four groups: control group (C group; *n* = 10; administered saline), romosozumab group (R group; *n* = 10; administered romosozumab at 25 mg/kg), vitamin D_3_ group (VD group; *n* = 10; administered active vitamin D_3_ at 0.2 µg/kg), and romosozumab plus vitamin D_3_ group (R + VD group; *n* = 10; administered romosozumab plus vitamin D_3_). Romosozumab (Amgen Inc., Japan, Tokyo) was administered once a month, whereas active vitamin D_3_ (Rocaltrol, Kyowakirin, Inc., Japan, Tokyo) was administered twice a week. In addition, the sham group (*n* = 10) was prepared but not included in statistically analyses. The study design is presented in Fig. 1.

### Obtaining femurs and blood samples

Ten weeks after femoral osteotomy, the rats were euthanized by injecting 0.15 mg/kg medetomidine hydrochloride + 2.0 mg/kg midazolam + 2.5 mg/kg butorphanol into the peritoneum, followed by cervical dislocation. The operated right femoral bones were explanted, and the bones were separated from the stainless-steel wire before analysis. The left femoral bones, which had not been operated upon, were used to evaluate the effect of the romosozumab and active vitamin D_3_ combination therapy on the bones of OVX rats using microcomputed tomography (micro-CT) analysis.

### Soft X-ray analysis

The right femoral bones obtained at 10 week after treatment were photographed using the Softex X-ray machine (Softex CSM-2; Softex, Tokyo, Japan) with HS Fuji Softex film (Fuji Film, Tokyo, Japan) at 45 cm, 30 kV, and 15 mA for 20 s. The fusion was quantified using the anteroposterior (A-P) and lateral radiographs. Three independent, blinded observers scored the bone formation in each rat using a four-point scale. Fracture union was judged by visual assessment of the mineralized callus bridging the fracture line on the A-P radiographs (right side: 1 point; left side: 1 point) and lateral radiographs (anterior side: 1 point; posterior side: 1 point). Bone fusion was considered as > 2 points on the four-point scale. In addition, the Radiographic Union Scale in Tibial Fractures (RUST) score was used to assess all specimens. The RUST score is based on the presence or absence of a callus and a visible fracture line at a total of four cortices on the A-P and lateral radiographs; the minimum score is 4 points, which corresponds to a fracture that as not healed, whereas the maximum is 12 points, which corresponds to a healed fracture with all cortices bridged with a callus and without a fracture line. Kooistra et al. reported the reliability and validity of the RUST scale in human long bone [20].

### Histopathological analysis

All femur specimens were harvested and dissected to remove soft tissue. The specimens were immersed in 70% ethanol followed by in 99.5% ethanol for 24 h each. The specimens were then sequentially immersed in acetone, 99.5% ethanol, and 2-propanol for 1 day each. The next day (day 6 after harvesting), the bones were embedded in glycolmethacrylate and allowed to stand for 10 days. Finally, using a fully automated rotary microtome (Leica RM2255, Leica, Nussloch, Germany), the femurs were cut into 3-µm-thick sections and stained with toluidine blue.

### Fracture bone micro-CT analysis (operated side)

Bone micro-CT imaging was performed according to the guidelines [21]. The explanted femoral bones were scanned using a Sky-Scan 1172 (Bruker micro-CT, Kontich, Belgium) with a voxel size of 20 mm. Data were collected at 100 kV and 100 mA and reconstructed using the cone-beam algorithm. Each femoral bone was placed on the object stage, and sample scanning was performed over 180° rotation with an exposure time of 105 ms. A cylindrical volume of interest with a diameter of 20 mm and a height of 27 mm was selected, which displayed the microstructure of the rat femoral bones (comprising the cortical and trabecular bones). Data analysis was performed using CT Analyzer (Bruker micro-CT). The area of fracture healing was considered the region of interest, which was defined by the fracture area filled with new bone; the structural indices of the femoral fracture areas (9 × 9 × 8.4 mm; a fracture gap in the center) were calculated using this software. In the three-dimensional analysis, the bone volume/tissue volume (BV/TV), BV of trabecular bone, TV, trabecular thickness (Tb. Th), trabecular number (Tb. N), and trabecular separation (Tb. Sp) were measured.

### Bone micro-CT analysis (nonoperated side)

Bone micro-CT imaging was performed according to the guidelines [21]. The explanted femoral bones were scanned using Sky-Scan 1172 (Bruker micro-CT, Kontich, Belgium) with a voxel size of 20 mm. Data were collected at 100 kV and 100 mA and reconstructed using the cone-beam algorithm. Each femoral bone was fixed in a sample holder and set with the vertical axis on the object stage, and sample scanning was performed over 180° of rotation with an exposure time of 105 ms. A cylindrical volume of interest with a diameter of 20 mm and a height of 27 mm was selected, which displayed the microstructure of the rat femoral bones (comprising the cortical and trabecular bones). Data analysis was performed using CT Analyzer software (Bruker micro-CT). The distal femoral area was considered the region of interest, and the structural indices of the femoral area of trabecular bone analysis (0.8–3.8 mm) from the growth-plate reference level and the area of cortical bone analysis (3.0–3.8 mm) from the growth-plate reference level were calculated using this software. During the three-dimensional analysis, the BV/TV; BV of trabecular bone; TV, Tb. Th, Tb. N, Tb. Sp; BV of cortical bone; cortical bone area (Cr. Ar); and cortical bone thickness (Cr. Th) were measured.

### Biomechanical analysis

Nonfractured femurs were used to determine bone strength. Three-point bending tests were conducted using a universal material testing system (Instron 5865; Instron, Kanagawa, Japan). The femur was placed on the sample support with its anterior surface facing upward and the center of the femoral shaft located at the center of the support. Load was applied at a rate of 1 mm/s until the bone fractured. An Instron program was used for data analysis, and the parameters of maximum load, maximum bending stress, stiffness, Young’s modulus, and toughness were measured.

### Measurement of serum bone turnover marker levels

Blood samples (100 µl) were collected when the animals were euthanized at week 10. Serum levels of osteocalcin (OC), an osteogenesis marker, were measured using a rat osteocalcin enzyme-linked immunosorbent assay (ELISA) kit (RK03858, Woburn, MA). The serum levels of cross-linked C-telopeptides of type-1 collagen (CTX-1), a bone-resorption marker, were measured using the rat cross-linked C-telopeptide of type-1 collagen ELISA kit (RK03603, Woburn, MA).

### Statistical analyses

Statistical analysis of all groups excluding the sham group was performed. Normality was confirmed using the Shapiro–Wilk test. Variables that were normative were subjected to one-way analysis of variance to compare the differences between the four groups. One-way analysis of variance with the Bonferroni post hoc test was used for the four-point test results, RUST, morphometric analysis, biomechanical analysis, and bone turnover marker analysis. For the four-point test and RUST, kappa statistics were performed to check for interobserver variability. The kappa statistic corrects the observed agreement for a possible chance agreement among observers. Agreement was rated as follows: *κ* = 0–0.20, poor; *κ* = 0.21–0.40, fair; *κ* = 0.41–0.60, moderate; *κ* = 0.61–0.80, substantial; and *κ* > 0.81, excellent. A value of 1 indicated absolute agreement, whereas a value of 0 indicated agreement no better than chance. All analyses were performed using SPSS V25.0 (IBM SPSS Statistics for Windows, IBM Corp., Armonk, NY). Statistical significance was set at *p* < 0.05.

## Results

### Body weight

**Fig. 2:**
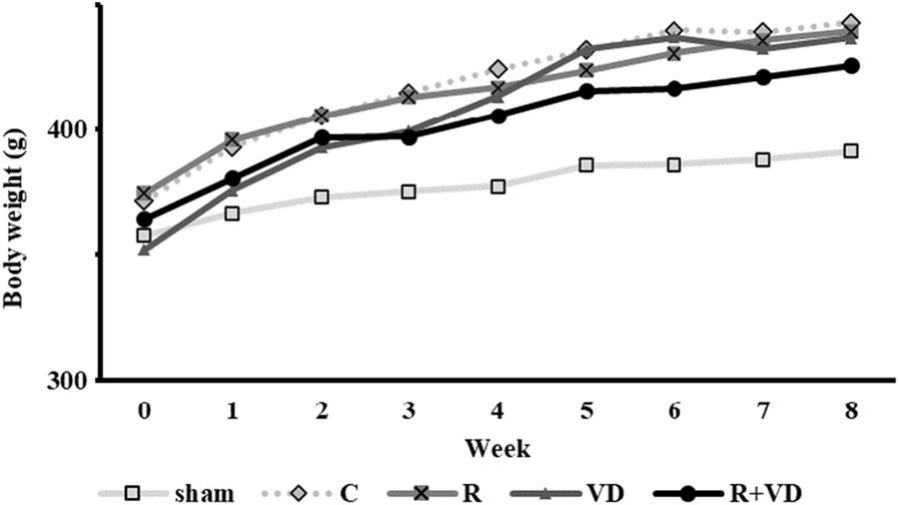
Mean body weight from ovariectomy to femoral surgery. Sham, sham group; C, control group; R, romosozumab group; VD, active vitamin D_3_ group; and R + VD group, romosozumab + active vitamin D_3_ group. The body weight of each rat was measured once a week for 8 weeks after ovariectomy. There was no significant difference among the four groups during the 8 weeks except in the sham group

The body weight of each rat was measured once a week for 8 weeks after ovariectomy. The results are presented in Fig. 2. No significant difference was observed of all groups excluding the sham group.

### X-ray imaging revealed no effect of the combination therapy on bone healing

Soft X-ray imaging of the femurs harvested at week 10 showed unclear fracture lines in the R, VD, and R + VD groups and abundant callus formation in the R and R + VD groups. In contrast, fracture lines were clear in the C group. Consistent agreement (*κ* = 0.88) was noted among the three independent observers who scored the radiographs. On the four-point scale, the C group scored 0.7 ± 0.8 points, the R group scored 0.4 ± 0.5 points, the VD group scored 1.3 ± 1.9 points, and the R + VD group scored 2.3 ± 1.6 points. There were no significant differences in the scores of the groups. On the RUST scale, the C group scored 8.0 ± 0.8 points, the R group scored 7.4 ± 1.3 points, the VD group scored 6.7 ± 1.3 points, and the R + VD group scored 7.9 ± 0.9 points. There were no significant differences in the scores of the groups (Table 1).

**Table 1:** Bone radiographic findings of rat femur and radiographic statistical analysis

	Sham	C	R	VD	R + VD
					
Four-point scale (point)	0.75 ± 1.5	0.7 ± 0.8	0.4 ± 0.5	1.3 ± 1.9	2.3 ± 1.6
RUST (point)	8.4 ± 1.4	8.0 ± 1.0	7.4 ± 1.3	6.7 ± 1.3	7.9 ± 0.9

Sham, sham group; C, control group; R, romosozumab group; VD, active vitamin D_3_ group; and R + VD, romosozumab + active vitamin D_3_ group. The fracture line was clear in the C group. In contrast, fracture lines were unclear in the R group, VD group, and R + VD group, whereas abundant callus formation was observed for the R and R + VD groups. No significant differences were observed in the four-point test and RUST scores between the groups

### Toluidine blue staining in nondecalcified specimens showed mature bridge formation without endochondral ossification in the R and R + VD groups

**Fig. 3:**
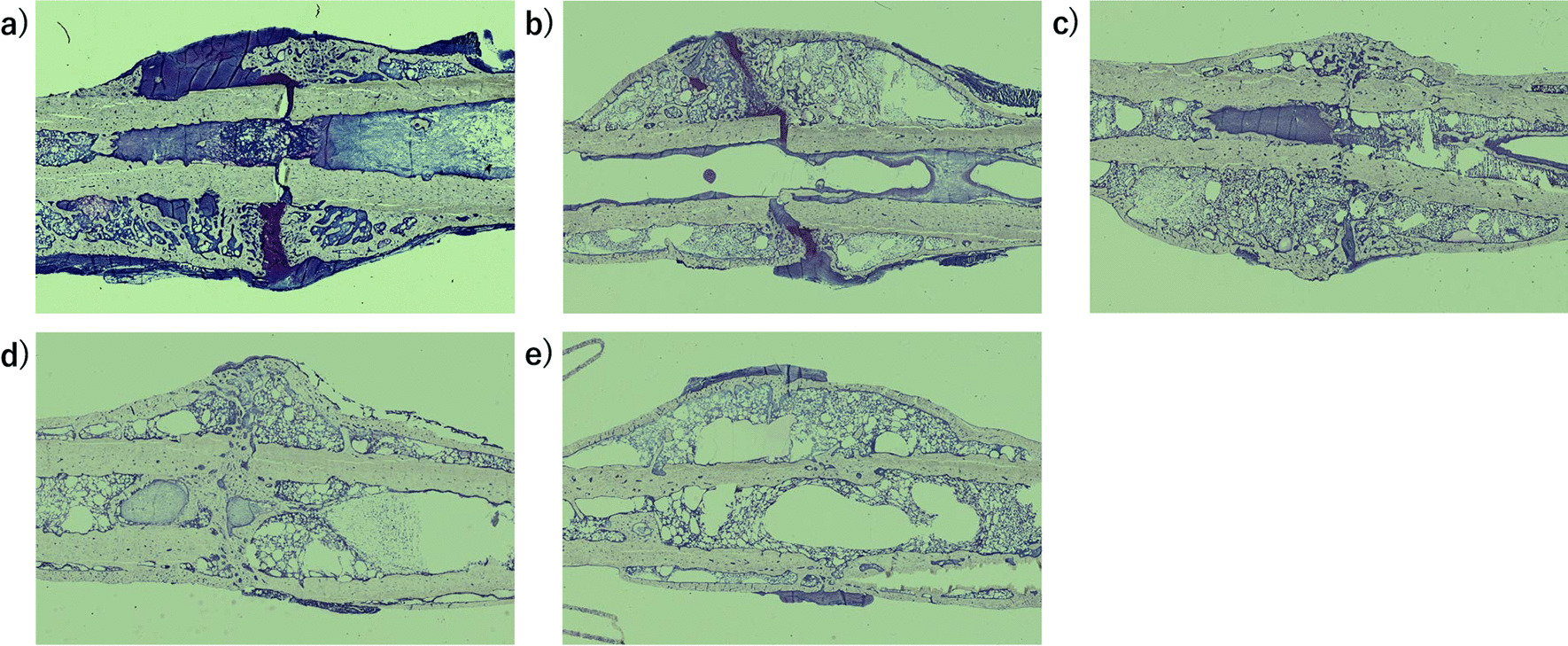
Results of bone histological analysis of the nondecalcified rat femur specimens (toluidine blue staining, × 1). **a** Sham group, **b** C group, **c** R group, **d** VD group, **e** R + VD group. Toluidine blue staining indicated immature callus formation and endochondral ossifications in the sham and C groups. However, mature bridges were formed without endochondral ossification in the R and R + VD groups

Bone histopathological analyses with toluidine blue staining indicated abundant immature callus formation and endochondral ossification in the sham and C groups. However, mature bridges were formed without endochondral ossification in the R and R + VD groups (Fig. 3).

### After combination therapy, micro-CT of the operated side showed no differences in all involved parameters

**Fig. 4:**
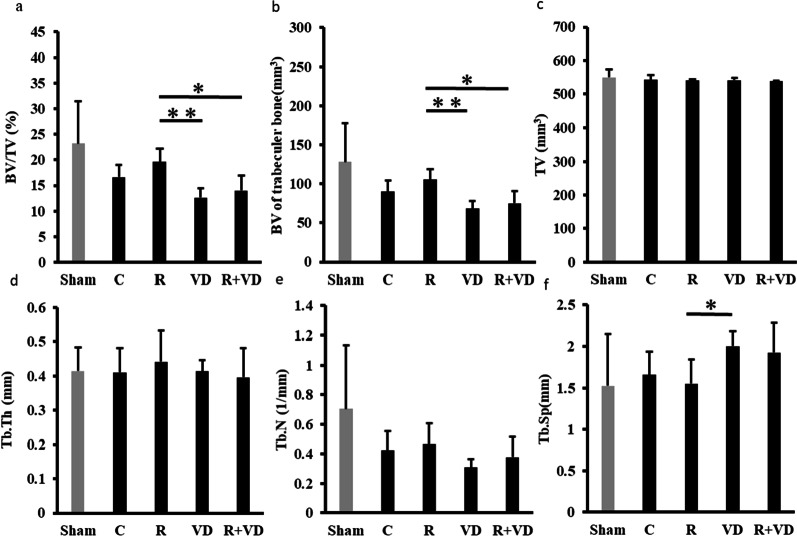
Results of bone morphometrical analysis of the operated side. **a** BV/TV: bone volume/tissue volume, **b** BV of trabecular bone, **c** TV, **d** Tb. Th: trabecular thickness, **e** Tb. N: trabecular number, **f** Tb. Sp: trabecular separation. The results of the morphological analysis of the femur on the fracture side are shown. BV of the trabecular bone in the R group was significantly higher than that in the VD group or R + VD group (*p* < 0.01, *p* < 0.05). Tb. Sp of the VD group was significantly higher than that of the R group (*p* < 0.01)

Results of micro-CT of the operated side are shown in Fig. 4. After the combination therapy, the microstructure parameters of the R + VD group were lower than that of the R group. The BV/TV and BV of trabecular bone were significantly higher in the R group than in the VD and R + VD groups (*p* < 0.01, *p* < 0.05). In contrast, BV/TV; BV of trabecular bone; and TV, Tb. Th, Tb. N, and Tb. Sp in the R and R + VD groups were not significantly different from those in the C group.

### After combination therapy, micro-CT of the nonoperated side showed absolute improvement in trabecular BV

**Fig. 5:**
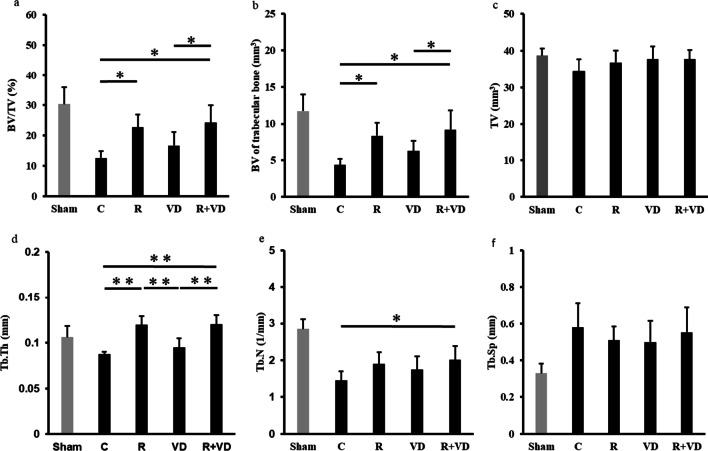
Results of trabecular bone morphometrical analysis of the nonoperated side. **a** BV/TV: Bone volume/tissue volume, **b** BV of trabecular bone, **c** TV, **d** Tb. Th: trabecular thickness, **e** Tb. N: trabecular number, **f** Tb. Sp: trabecular separation. BV/TV and BV of the trabecular bone were significantly higher in the R and R + VD groups than those in the C group (*p* < 0.05, *p* < 0.01). Tb. Th and Tb. N were significantly higher in the R and R + VD groups than in the C group

Results of micro-CT of the nonoperated side are shown in Fig. 5. BV/TV and BV of the trabecular bone were significantly higher in the R and R + VD groups than in the C group (*p* < 0.05, *p* < 0.01). Compared with the C group, significant differences were observed in the Tb. Th and Tb. N of the R and R + VD groups, indicating increases in Tb. Th and Tb. N and a decrease in Tb. Sp. No significant differences were observed in the cortical bone for all groups (Table 2).

**Table 2:** Results of cortical bone morphometrical analysis for the nonoperated side

	Sham	C	R	VD	R + VD
					
BV of cortical bone (mm^3^)	4.65 ± 0.46	4.87 ± 0.25	5.05 ± 0.26	4.65 ± 0.34	5.15 ± 0.39
Cr. Ar (mm^2^)	5.68 ± 0.56	5.96 ± 0.30	6.18 ± 0.32	5.69 ± 0.42	6.29 ± 0.48
Cr. Th (mm)	0.36 ± 0.04	0.36 ± 0.02	0.39 ± 0.03	0.34 ± 0.03	0.38 ± 0.04

Sham, sham group; C, control group; R, romosozumab group; VD, active vitamin D_3_ group; and R + VD, romosozumab + active vitamin D_3_ group. Cr. Ar, cortical bone area; Cr. Th, cortical bone thickness. All parameters showed a greater increase in the R and R + VD groups than in the C group, but no significant differences were observed in BV, area, and thickness of the cortical bone between the groups

### The three-point bending test showed that combination therapy increased bone strength

**Fig. 6:**
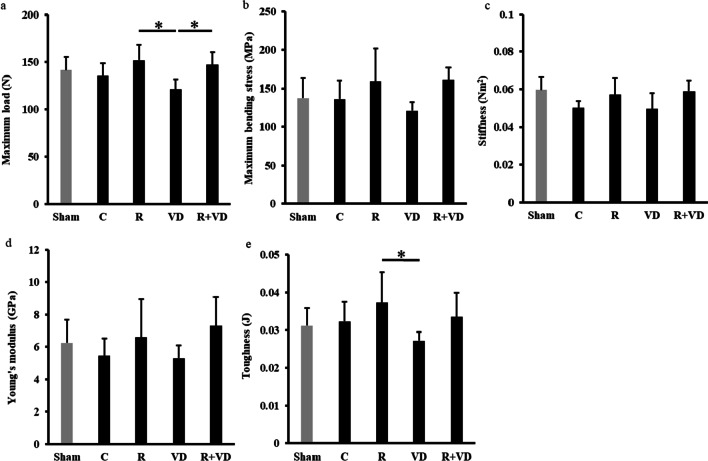
Results of biomechanical analysis of the nonoperated side. **a** Maximum load, **b** maximum bending stress, **c** stiffness, **d** Young’s modulus, **e** toughness. The values of maximum bending stress, stiffness, and Young’s modulus were higher in the order of R + VD group > R group > C group. The values of maximum load and toughness were higher in the order of R group > R + VD group > C group

The three-point bending test results are presented in Fig. 6. The values of maximum bending stress, stiffness, and Young’s modulus were higher in the order of R + VD group > R group > C group. The values of maximum load and toughness were higher in the order of R group > R + VD group > C group.

### Bone turnover markers were comparable between all groups

The OC and CTX-1 measurement results are presented in Table 3. No significant differences in the serum OC and CTX-1 levels of the groups were noted.

**Table 3:** Results of serum bone turnover marker level analysis

	Sham	C	R	VD	R + VD
OC (pg/mL)	126.29 ± 45.94	110.15 ± 51.33	111.76 ± 25.13	136.30 ± 55.66	175.28 ± 125.34
CTX-1 (pg/mL)	443.97 ± 69.31	471.74 ± 56.94	473.94 ± 51.30	478.47 ± 44.61	456.92 ± 53.79

Sham: sham group, C: control group, R: romosozumab group, VD: active vitamin D_3_ group, R + VD: romosozumab + active vitamin D_3_ group. OC: osteocalcin, CTX-1: cross-linked C-telopeptide of type-1 collagen. Osteocalcin (OC) showed bone-formation activity, and cross-linked C-telopeptide of type-1 collagen (CTX-1) showed bone-resorption activity. The OC values were higher in the VD and R + VD groups compared with the C group. The CTX-1 values in every group were similar. No significant differences in the serum levels of OC and CTX-1 were noted between the groups.

## Discussion

Evaluation of the operated side revealed that romosozumab and active vitamin D_3_ combination therapy did not increase callus mass or improve cortical bone continuity. Previous reports on romosozumab in rats used romosozumab at a dose higher than the conversion dose for humans. It is also unclear whether previous studies investigated the true effects of romosozumab. Therefore, in the present study, we used a dose of 25 mg/kg once a month. The results of the nonfracture side proved that romosozumab was sufficiently effective at that dose. Suen et al. indicated that treatment with romosozumab (25 mg/kg, twice a week) significantly increased the formation of new blood vessels in a rat fracture model [14]. Feng et al. showed that treatment with romosozumab (25 mg/kg, twice a week) increased the level of bone morphogenetic protein-2, a bone morphogenetic factor, in an 8-week-old OVX rat fracture model [13]. These results imply that romosozumab has a bone union-promoting effect. However, Morse et al. reported that romosozumab treatment (25 mg/kg biweekly) increased callus formation and bone strength but not the bone union rate in a rat fracture model [15]. This is consistent with our results. In addition, there are two clinical trials about the efficacy of acceleration of bone fracture healing with romosozumab in adult fresh fractures. Schemitsch et al. reported a phase II clinical trial of romosozumab for the treatment of hip fractures [22] wherein no significant difference was noted between the treatment groups. Bhandari et al. reported the efficacy of romosozumab in fresh unilateral tibial diaphyseal fractures with intramedullary nail fixation [23]. There was no significant difference in the bone union rate and time to clinical healing between romosozumab group and placebo group. Consistent with the results in patients with hip fractures, romosozumab did not improve fracture healing in patients with tibia fractures.

The results of micro-CT of the femur on the nonfractured side demonstrated that romosozumab treatment improved the microstructure of the trabecular bone. Increases in trabecular bone mass, Tb. Th, and Tb. N on the nonfractured side were found in the rats receiving romosozumab and active vitamin D_3_ combination therapy, thus confirming that the morphological changes were equivalent or greater than those obtained with romosozumab treatment alone. Some reports have indicated that romosozumab treatment in OVX models has bone anabolic effects [24, 25], especially in terms of improvement in the loss of cancellous bone. The results of micro-CT of the nonfracture side showed that the effect of romosozumab plus vitamin D_3_ combination therapy was equal or superior to that of romosozumab treatment alone. However, the results of micro-CT of the fracture side, including BV/TV and trabecular bone BV, showed no advantage of the combination therapy over romosozumab treatment alone. Although the cause is unknown, the combination of vitamin D_3_ with romosozumab may not have a cooperative effect on the fracture side. In OVX rats, the administration of active vitamin D_3_ has been reported to significantly decrease the number of osteoclasts in bone tissue and affect osteoblast differentiation and remodeling [26]. Furthermore, romosozumab-mediated promotion of modeling-based bone formation was considered as the cause.

Regarding bone turnover, a study on humans showed that an increase in bone-formation marker levels and decrease in bone-resorption marker levels peaked on day 14 after romosozumab administration [27]. However, no such difference was observed in our study, presumably because the bone turnover marker levels were measured only when the animals were euthanized at week 10.

There are certain limitations to this study. For example, the progress of fracture healing was not followed over time using radiography and bone turnover marker levels. Preliminary studies showed no change during fracture treatment by X-ray and no difference in bone metabolic markers.

## Conclusions

OVX fracture model rats were subjected to romosozumab and active vitamin D_3_ combination therapy. The results indicated that the combination therapy improved trabecular BV but did not accelerate the fracture healing process. It appears that romosozumab treatment alone or romosozumab and active vitamin D_3_ combination therapy does not accelerate the fracture healing process. The effect of romosozumab on fracture healing remains unclear. Further studies must consider sclerostin expression during the early and late phases of fracture repair, including genetic and immunohistology perspectives, to elucidate the mechanism of action of romosozumab. As a result, alternative dosing strategies for fracture healing may be needed.

## Data Availability

All data generated or analyzed during this study are included in this published article.

## Abbreviations

cWnt: Canonical Wnt
LRP: Low-density lipoprotein receptor-related protein
OVX: Ovariectomized
RUST: Radiographic union scale in tibial fractures
BV/TV: Bone volume/tissue volume
Tb. Th: Trabecular thickness
Tb. N: Trabecular number
Tb. Sp: Trabecular separation
Cr. Ar: Cortical bone area
Cr. Th: Cortical bone thickness
OC: Osteocalcin
CTX-1: Cross-linked C-telopeptides of type-1 collagen
ELISA: Enzyme-linked immunosorbent assay
R: Romosozumab
VD: Active vitamin D_3_
